# Supplementary material for: Conditional chemoconnectomics (cCCTomics) as a strategy for efficient and conditional targeting of chemical transmission
Source: eLife. 2024 Apr 30;12:RP91927. doi: 10.7554/eLife.91927 (PMC11060718; doi:10.7554/eLife.91927)
Supplement: Figure 3—source data 3. [file elife-91927-fig3-data3.zip › Figure 3 source data3 part 1.zip!/Figure 3-Source data 3-part1/1071-1-CRISPResso_Report_osfbjvga.zip!/CRISPResso_on_1071_1/1c.Alignment_barplot.pdf]

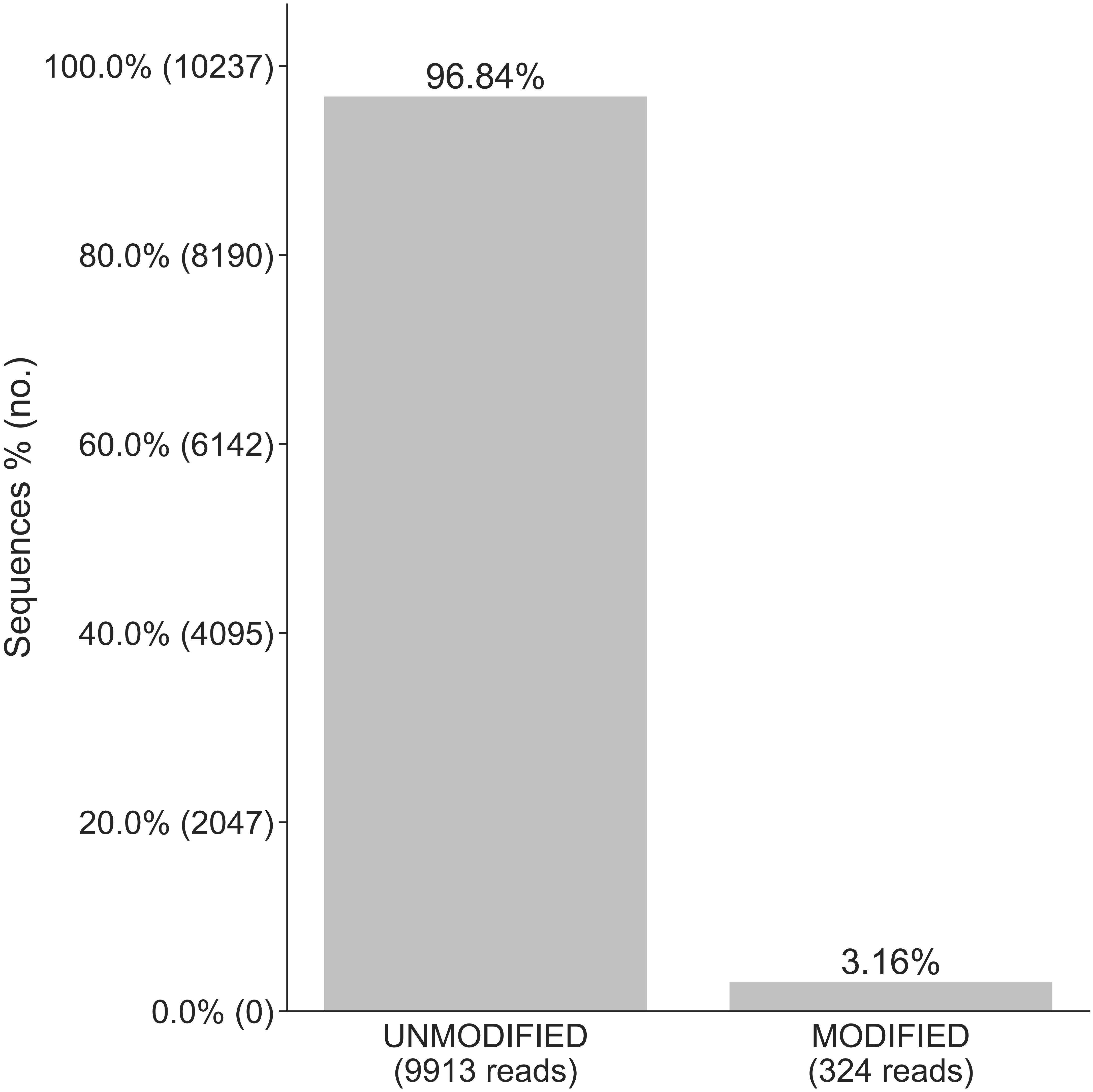

Sequences % (no.)
100.0% (10237)
80.0% (8190)
60.0% (6142)
40.0% (4095)
20.0% (2047)
0.0% (0)
96.84%
3.16%
UNMODIFIED
(9913 reads)
MODIFIED
(324 reads)
